# A Plea for the Dumb Animals

**Published:** 1886-11-06

**Authors:** Anna Drury


### A PLEA FOR THE DUMB ANIMALS.

St. James ii, 13.

YE call them "dumb," and deem it well,
Howe'er their bursting hearts may swell,
They have no voice their woes to tell,
As fabulists have dreamed.
They cannot cry, "O Lord, how long
Wilt Thou, the patient Judge and strong,
Behold Thy creatures suffer wrong
Of those Thy Blood redeemed?"

Yet are they silent? Need they speech
His holy sympathies to reach,
Who by their lips could prophets teach,
And for their sakes would spare;
When, wrestling with His own decree,
To save repentant Nineveh,
He found, to strengthen mercy's plea,
So many cattle there?

Have they no language? Angels know,
Who take account of every blow;
And there are angel hearts below
On whom the Eternal Dove
His pentecostal gift hath poured,
And that forgotten speech restored
That filled the Garden of the Lord
When Nature's voice was Love!

Oh, blest are they the creatures bless!
And yet that wealth of tenderness
In look, in gesture, and caress,
By which our hearts they touch,
Might well the thoughtful spirit grieve,
Believing—as we must believe—
How little they from man receive,
To whom they give so much!

They may be silent, as ye say,
But woe to them who day by day,
Unthinking for what boon they pray,
Repeat, "Thy Kingdom Come":
Who, when before the Great White Throne,
They plead that Mercy may be shown,
Find awful voices drown their own—
The Voices of the Dumb!

ANNA DRURY.

